# Integrating Models of Diffusion and Behavior to Predict Innovation Adoption, Maintenance, and Social Diffusion

**DOI:** 10.1080/10810730.2018.1434259

**Published:** 2018-02-15

**Authors:** Rachel A Smith, Youllee Kim, Xun Zhu, Dimi Théodore Doudou, Eleanore D. Sternberg, Matthew B. Thomas

**Affiliations:** 1 Department of Communication Arts and Sciences, The Pennsylvania State University, University Park, PA, USA; 2 Centre de recherche pour le Développement (CRD)/Laboratoire de Santé, Nutrition et Hygiène, Université Alassane Ouattara, Bouaké, Côte d’Ivoire; 3 Department of Entomology, The Pennsylvania State University, University Park, PA, USA

## Abstract

This study documents an investigation into the adoption and diffusion of eave tubes, a novel mosquito vector control, during a large-scale scientific field trial in West Africa. The diffusion of innovations (DOI) and the integrated model of behavior (IMB) were integrated (i.e., innovation attributes with attitudes and social pressures with norms) to predict participants’ (*N* = 329) diffusion intentions. The findings showed that positive attitudes about the innovation’s attributes were a consistent positive predictor of diffusion intentions: adopting it, maintaining it, and talking with others about it. As expected by the DOI and the IMB, the social pressure created by a descriptive norm positively predicted intentions to adopt and maintain the innovation. Drawing upon sharing research, we argued that the descriptive norm may dampen future talk about the innovation, because it may no longer be seen as a novel, useful topic to discuss. As predicted, the results showed that as the descriptive norm increased, the intention to talk about the innovation decreased. These results provide broad support for integrating the DOI and the IMB to predict diffusion and for efforts to draw on other research to understand motivations for social diffusion.

*So the opinion leader must continually look over his or her shoulder, and consider where the rest of the system is at regarding new ideas.*

— (Rogers, 2003), p. 296

In 2015, over 212 million people were sick with malaria worldwide (90% in Africa), resulting in 429,000 deaths (WHO, 2016). Even after decades of intervention, few countries have eliminated malaria (World Health Organization, 2016). Much effort has focused on controlling the mosquito vectors responsible for malaria transmission, such as reducing the likelihood of being bitten by an infectious mosquito, killing mosquitos where they rest, and reducing reservoirs for new mosquitoes (Keiser, Singer, & Utzinger, 2005). These approaches have limitations. For example, mosquitos can bite when people are not sleeping (Guerin et al., 2002), and mosquitoes have shown increased resistance to existing insecticides (Ranson & Lissenden, 2016). New tools for malaria prevention are needed in many settings (Barreaux et al., 2017).

Development of a new mosquito-control intervention involves extensive field-testing in order to demonstrate efficacy and obtain a recommendation from the World Health Organization. However, efficacy is only part of the challenge; to address the problem, end users must adopt the innovations. This study investigated the potential adoption and diffusion of a novel vector control, referred to as the Screening and Eave Tubes (SET) innovation, during a large-scale scientific, field-trial in West Africa. The diffusion of innovations (DOI; Rogers, 2003) and the integrated model of behavior (IMB; Fishbein, 2000; Fishbein & Cappella, 2010) were used to anticipate its potential adoption, maintenance, and social diffusion.

## Understanding the Adoption of Innovations

For over a century, scholars have been documenting the adoption of innovations and considering their predictors (Dearing, 2008). The studies have typically been retrospective (Dearing, 2008), documenting successes and failures (see Rogers, 2003). Recently, there has been a growing emphasis on predictive studies in which scholars use the DOI “to accelerate the pace of adoption, increase the number of adoptions, enhance the quality of innovation implementation, sustain the use of worthy innovations, and, as ultimate outcomes, demonstrate innovation effectiveness at individual client and client system levels” (Dearing, 2008, p. 99).^1^ 1In some disciplines, predictive studies have incorporated different terms, for example, using *dissemination* to refer to intentional, strategic efforts to spread innovations, but *diffusion* for unintentional spread (Green, Ottoson, Garcia, & Haitt, 2009; Rabin, Brownson, Haire-Joshu, Kreuter, & Weaver, 2008). Sometimes, the use and adaptation of innovations by end users have been referred to as implementation research (Green et al., 2009). In this paper, we used Rogers’ (2003) inclusive definition of diffusion, which included adoption, utilization, re-invention (i.e., adaptation by end users), and spread (intended or not).


Innovations are ideas, practices, or objects that adopters (people, groups, companies or other social agents) perceive to be novel (Rogers, 2003). The element of novelty creates uncertainty (Rogers, 2003). Diffusion results from reducing uncertainty about the innovation through conversations with people to gain insights into reasons for adoption, such as desirable attributes of the innovation and social pressures to adopt it (Dearing, 2008; Rogers, 2003). All else being equal, innovations with more desirable attributes and greater social pressure to adopt them are more likely to be adopted. However, these predictors vary across people (Rogers, 2003). Innovators are the first to adopt; for them, the risk of uncertain, novel ideas is a draw. Others adopt because they like the innovation’s attributes, or because they want to adopt what others, especially opinion leaders, have adopted. Laggards, who are deeply risk-adverse, are the last to adopt (Rogers, 2003). The likelihood of widespread adoption, then, results from personal traits, innovation attributes, and social pressure.

Campaigns attempting to harness the power of diffusion processes could identify innovators and opinion leaders within the end-user population and then create messages that persuade them (a) to adopt the innovation and (b) to encourage others to adopt it (Dearing, 2008; Rogers, 2003; Smith & Findeis, 2013). In practice, however, it is quite difficult to identify opinion leaders and innovators.

An alternative is to adjust an innovation’s attributes to improve its adoption. Often, diffusion efforts start after an innovative technology’s design is finished (Rogers, 2003; Smith & Findeis, 2013). Including social science research into the field-testing phases offers an opportunity to identify how end users perceive an innovation and discuss it with others. Innovation designers could use this information as they make final design decisions. Campaigners can use the same information to anticipate potential obstacles to adoption and to design diffusion strategies with these obstacles in mind.

## Using DOI and IMB to Predict Diffusion

To predict diffusion, we integrated predictions from the DOI (Rogers, 2003) with a robust theory of behavior: the integrated model of behavior (IMB; Fishbein, 2000; Fishbein & Ajzen, 2010). Three key components of diffusion – adoption, utilization, and interpersonal conversations about the innovation – are all behaviors, which fits in the scope of the IMB. In addition, the three predictors highlighted in the DOI – innovation attributes, social pressure, and personal traits–align with predictors of behavioral intentions in the IMB. The IMB states that, presuming no environmental or skill constraints, the best predictor of behavior is one’s intention to do it, and that intention is predicted by attitudes toward the behavior, social norms encouraging it, and the efficacy to perform it.

Self-efficacy was less relevant in our setting. The innovation was offered to participants for free, and was installed by professionals, and the research team visited the homes regularly to verify installation and maintenance. Therefore, we focused on integrating the attitudes and norms variables from the IMB with the attributes and social pressures variables from the DOI.

### Attitudes: Innovation Attributes

The first integration between the IMB and the DOI was to use judgments of the innovation’s attributes as the relevant attitudes to predict behavior. In the IMB, attitudes are evaluations of “a psychological object” (Fishbein & Azjen, 2010, p. 76). Rogers (2003) outlined five perceptions of an innovation’s attributes that influence adoption: relative advantage, compatibility, complexity, trialability, and observability. Relative advantage is the perception that the innovation is better (in terms of economics, social prestige, convenience, or satisfaction) than what currently exists. Compatability is the perceived match between the innovation and adopters’ values, past experiences, and needs. Complexity is the perceived difficulty in understanding and using an innovation. Trialability is perceived ability to experiment with the innovation before adoption. Observability is the perceived visibility, specifically that the “results of an innovation are visible to others” (Rogers, 2003, p. 16). Some innovative technologies inherently constrain the degree to which these perceptions can vary. The SET innovation is no exception.

### The SET Innovation

**Fig. 1.**
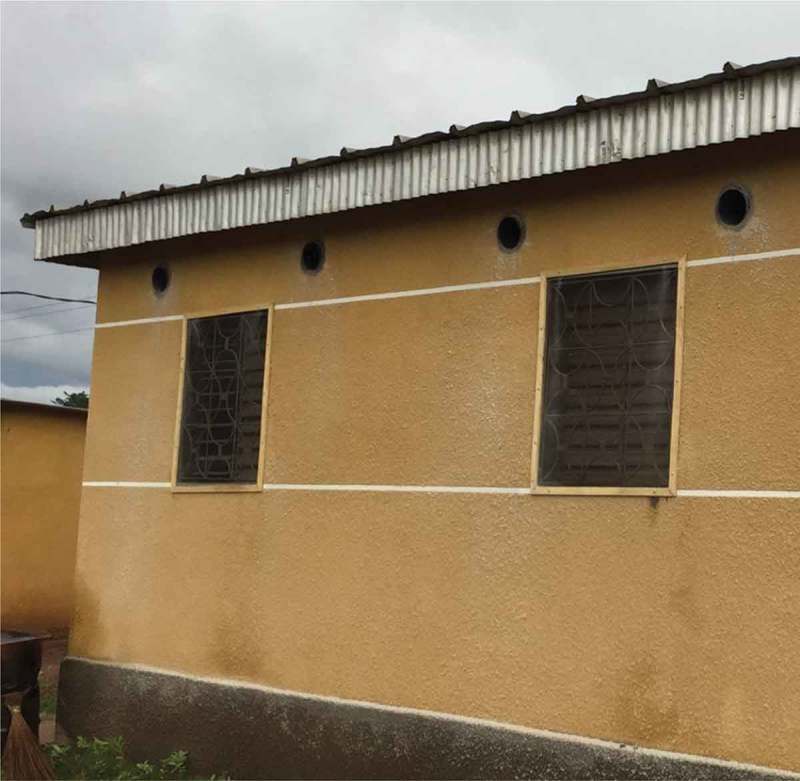
An example of the SET innovation that includes closing points of entry for mosquitoes into the home (e.g., closing eaves in houses where they remain open), adding mosquito barriers to windows and doors (e.g., installing window screening), installing tubes into the eaves, and placing an insert with insecticide into the tubes.

In 2012, a diverse group of researchers developed a novel means to reduce people’s exposure to bites from mosquitoes that carry the malaria parasite (Knols et al., 2016). They used insights into mosquito and human behavior to improve household protection against mosquitoes and to create a new system for delivering insecticides. In brief (for more details, see Knols et al., 2016), infectious mosquitos are predominantly nocturnal and feed indoors. Housing in Africa is changing to include more durable materials (e.g., from thatch to metal roofs) and to seal open eaves (the space between the top of the wall and the roof). Although these changes do reduce mosquitoes’ entry into homes (Tusting et al., 2015), they also created issues with heat and ventilation in homes, which generally do not have cooling systems. The researchers created a suite of modifications, referred to as the SET innovation, which includes closing points of entry for mosquitoes into the home (e.g., closing eaves in houses where they remain open), adding mosquito barriers to windows and doors (e.g., installing window screening), installing tubes into the eaves, and placing an insert with insecticide into the tubes (see Figure 1). The eave tubes provide air circulation, ambient light, and a conduit for people’s odors to exit the house. Mosquitos are drawn to those odors, and then make contact with the insecticide in the tube (Knols et al., 2016). As such, the approach essentially turns the house into a ‘lethal lure’ that not only reduces the entry of mosquitoes but also kills them as they search around the house and attempt to enter via the eave tubes (Sternberg et al., 2016). This mortality is predicted to provide community protection once the coverage of the SET innovation reaches a certain level (i.e., screening alone ought to benefit individual householders, but by killing mosquitoes the benefit of SET should extend also to householders who have not adopted the technology) (Waite, Lynch, & Thomas, 2016). Once installed, the SET innovation requires little upkeep. Homeowners need to monitor that the eave tubes are not blocked by debris, change the insecticide inserts periodically (which was done for them in the trial), and repair holes or cracks in the house modifications (e.g., a hole in the screen).

The SET innovation, then, was intended to have relative advantages over existing housing methods to limit indoor mosquitoes, high compatibility with needs for indoor comfort, and low complexity. The SET innovation does not have trialability: The house modifications and installation of the eave tubes are difficult to undo. The innovation may also have limited observability. People are unlikely to see the mosquitos dying or to find dead mosquitos, because the mosquitoes typically fly off before they die. However, the innovation suite itself is visible: the sealed walls and eave tubes are visible when walking by a home. The innovation may be perceived as making the house more attractive. If the designers’ intentions worked, then end-users should judge the innovations attributes positively; this should result in diffusion.

H1: More positive attitudes about the SET innovation’s attributes predict greater intentions to adopt the innovation and maintain it.

### Norms as Social Pressure

The second integration focused on social pressure and norms. In the IMB (Fishbein, 2000; Fishbein & Ajzen, 2010), people’s perceptions of what other people are doing–descriptive norms–are powerful predictors of behavior. People are inclined to do what others are doing. In DOI (Rogers, 2003), perceiving that others are adopting an innovation reduces the uncertainty associated with novel ideas. In other words, one adopts because others are adopting. The concept and influence of social pressure created by descriptive norms on behavior is the same in both theories.

H2: A stronger descriptive norm that others will adopt the SET innovation predicts greater intentions to adopt and maintain the innovation.

### Personal Trait to Adopt Early

The last integration is to include the individual trait of being the kind of person who likes to adopt novel ideas, which is a key component of DOI (Rogers, 2003). In the IMB, such traits are considered distal predictors of behavioral intentions that are mediated by attitudes and social norms. Tests of the IMB have typically focused on global personality features (e.g., introversion and extroversion; Fishbein & Ajzen, 2010). In the DOI, the inclination to adopt before others, however, is considered as a direct predictor and even a moderator that shapes whether attitudes or norms are more important; those who adopt sooner are argued to be persuaded by attributes, and the later adopters by social pressures (Dearing, 2008). We retained the DOI’s prediction that the trait has a direct effect on behavior, and we explored possible moderation.

H3: A greater tendency to adopt innovations before others predicts greater intentions to adopt and maintain the innovation.

RQ1: Are the predictors different for earlier versus later adopters?

## Social Diffusion: The Role of Conversation

As stated earlier, Rogers (2003) considered diffusion as a fundamentally social phenomenon. Through interpersonal conversations, people become aware of innovations, develop beliefs about innovations’ attributes, and form perceptions about other people’s likelihood of adopting them. The field trial’s protocol included a town hall meeting with the community to tell them about the trial and the SET innovation. People who attended that meeting and talked about it with others should have had a greater store of relevant information about the innovation suite. All else being equal, diffusion is faster among those with a greater store of relevant information and who talk about it with others (Rogers, 2003). Conversation does not just benefit the sharer: those who heard about the SET innovation from others who attended the town hall meeting (through word-of-mouth) also may have reduced uncertainty about the innovation and be more likely to adopt it.

H4: Sharing or hearing about the Town hall meeting through word of mouth predicts greater intentions to adopt and maintain the innovation.

In his review of research into how interpersonal interactions affect public understanding and reactions to science, Southwell (2017) argued that more work is needed to investigate whether people talk about the science they hear about and also what they decide to share and what their listeners hear.

RQ3: What do people decide to share and remember hearing about the SET innovation?

### Discussions as a Dependent Variable

The role of conversation cannot be overestimated in diffusion: it is a critical means by which end-users reduce their uncertainty about an innovation’s attributes and others’ uptake of it (Rogers, 2003; see also Southwell & Yzer, 2009). Although it is useful to see how word-of-mouth about a one-to-many town hall meeting predicts adoption, it is as important to predict end-users’ intentions to discuss the innovation with others.

From the DOI and the IMB, it is not clear whether attitudes about the innovation’s attributes and social pressure created by descriptive norms will predict future conversation. Research into sharing finds that people have a bias toward sharing novel and useful information (Berger, 2014; Cappella, Kim, & Albarracín, 2015). Doing so makes the sharer feel (Cappella et al., 2015) and appear to others like they are smart, interesting, and “in the know” (Berger, 2014, p. 591). Thus, people may talk with others about an innovation to make a good impression. People who adopt earlier than others may be particularly inclined to discuss the topic with others, because it can reinforce their innovativeness. The descriptive norm of adoption, however, may dampen sharing: as the perception that others are adopting the innovation increases, it may be seen as a less novel, useful topic.

H5: Weaker descriptive norms, more positive attitudes toward the SET innovation, greater identification as an early adopter, and talking and hearing about the town hall predict stronger intentions to talk about the innovation with friends and family.

## Method

### Participants

Forty villages were selected for the larger, randomized, control trial of the SET innovation’s efficacy (see supplementary material for details). Six villages were selected at random to participate in this study of end users’ adoption. The survey was restricted to heads of households who owned the property of their residence and could decide whether to adopt the SET innovation. Property owners often own more than one home and rent to other families. Across the six villages, 333 property owners were identified; 329 property owners (99% response rate) agreed to participate in the study and constituted our final sample. On average, each village had 55 property owners (*SD* = 14.41, *minimum* = 37, *maximum* = 77).

The property owners (71% male) on average were 60 years old (*SD* = 14.64, *Mdn* = 60, *minimum* = 24, *maximum* = 115). Their households often included multiple adults and children: 5 adults over the age of 21 (*SD* = 2.88, *Mdn* = 4, *minimum* = 1, *maximum* = 17) and five youth 21 or younger (*SD* = 4.53, *Mdn* = 5, *minimum* = 0, *maximum* = 35). In the past year, on average, 32% of adults and 49% of youth were sick with malaria. About 17% of the property owners reported being sick with malaria, confirmed by a doctor, in the past 2 weeks. Most property owners (80%) reported sleeping under a bednet the previous night. Property owners reported having no formal schooling (65%), having attended primary school (18%), secondary school (15%), or post-secondary school (2%). Most were not at all comfortable reading (65%) or writing (66%) in French; they all were comfortable with a local language, Baoulé.

### Procedures

Details on the trial’s protocol, village selection, survey translation and interviewer training are available in the online supplement. After identifying property owners, interviewers explained and discussed the study. After gaining consent, interviewers recorded participants’ answers on tablet computers. Two supervisors were present during the survey process; to oversee the surveying process and address technical difficulties.

### Measurement

Across items, 17 out of the 329 participants had one missing item. The missing data were replaced with mean scores (Anderson, Basilevsky, & Hum, 1983). A confirmatory factor analysis of the measurement model’s scales ‒ attribute attitudes, descriptive norms, earlier adopter, and the intention to maintain the SET innovation ‒ was estimated with maximum likelihood in AMOS (Version 24). The four latent factors were allowed to covary, but the error terms were not. The measurement model showed reasonable fit: *χ* ^2^(146, *N* = 329) = 364.06, *p* < .05, SRMR = .07, RMSEA = .07, 90% CI [.06, .08]. Higher scores indicate more of the variable.

#### Attribute Attitudes

Ten items (created based on Rogers, 2003) were used to assess attitudes about the innovation’s attributes (e.g., the SET innovation is better than other malaria prevention technologies, is safe for children, fits with my lifestyle, will make my house prettier). Responses, marked on 3-point scales (1 = *disagree*, 3 = *agree*), were averaged into one score (Cronbach’s *α* = .78).

#### Descriptive Norm

Three items were used to assess the degree to which participants anticipated that (a) people in the community, (b) important others, and (c) community leaders would adopt the SET innovation. Responses, marked on 11-point scales (0 = *none of them*, 10 = *all of them*), were averaged into one score (Cronbach’s *α* = .82).

#### Town Hall Shared and Word-Of-Mouth (WOM)

Participants were asked whether they attended the town hall meeting. Those who attended (*n* = 121, 37%) were asked whether they discussed what they heard at the meeting with others. About half of the attendees discussed it with others (*n* = 57, 17% of total); an effect code (i.e., *town hall shared*) was created (1 = *attended and discussed the town hall meeting*, −1 = *everyone else*). Participants who shared about the town hall were presented an open-ended question in which they were asked what they told people. Those who did not attend the meeting (*n* = 208, 63%) were asked if anyone ever spoke to them about the town hall meeting. About a third of those who did not attend the meeting heard about it from others through WOM (*n* = 65, 20% of total); an effect code (i.e., *Town hall WOM*) was created (1 = *heard about the town hall meeting through WOM*, −1 = *everyone else*). Town hall WOM participants were presented an open-ended question in which they were asked what other people told them.

#### Earlier Adopter

Three items were used to assess the degree to which participants tend to adopt new technologies before others. Responses, marked on 5-point scales (1 = *not at all*, 5 = *a lot*), were averaged into one score (Cronbach’s *α* = .87).

#### Economic Resources

Participants were asked whether they personally owned a radio, a television, a mobile phone, a moped and a car. The five answers were summed into a single score indicating how many items they personally owned.

#### Intention to Maintain and to Adopt

Three items (Cronbach’s *α* = .95) were used to assess participants’ intentions to keep the SET innovation in good working order (e.g., clearing away any debris weekly). A single item was used to assess participants’ intention to adopt the SET innovation. Responses were marked on 6-point scales (0 = *not at all*, 5 = *strongly intend*).

#### Intention to Talk

A single item was used to assess participants’ intention to talk about the SET innovation with their friends and family. The response was marked on a 3-point scale (1 = disagree, 3 = agree).

## Results

### Descriptive Statistics

Descriptive statistics appear in Table 1. On average, property owners had positive attitudes about the SET innovation, and anticipated that most people in their community, including community leaders, would adopt the SET innovation. Property owners varied in the degree to which they try innovations (like new technologies) before others; the average score was at the scale’s mid-point. On average, property owners intended to adopt, maintain, and talk about the SET innovation with other people. Of note, in one village ultimately assigned to treatment, a check was conducted to assess the association between participants’ intentions to adopt and actual adoption: of those who reported that they were very likely to adopt (5 on the 5-point scale), 90% agreed to have the SET innovation installed.

**Table 1.** Descriptive statistics and correlations among variables (*N* = 329)

	*M*	*SD*	1.	2.	3.	4.	5.	6.	7.	8.
1. Attribute attitudes	2.80	0.25	–							
2. Descriptive norm	8.84	1.62	.21**	–						
3. Town hall shared WOM	−0.65	0.76	.11	.03	–					
4. Town hall heard WOM	−0.60	0.80	−.03	.02	−.23**	–				
5. Earlier adopter	3.08	1.39	.01	.20**	.15**	.11*	–			
6. Economic resources	1.93	1.24	.10	.10	.09	.03	.07	–		
7. Intention to adopt	4.78	0.73	.23**	.32**	.05	−.18*	.11*	.17**	–	
8. Intention to maintain	4.82	0.52	.26**	.28**	.11	−.05	.04	.14**	.30**	–
9. Intention to talk	2.79	0.46	.59**	.07	.19**	.03	.11*	.11*	.15**	.24**

* *p* < .05, ** *p* < .01.

#### Non-Normality

Intention to adopt and intention to maintain were negatively skewed (*skew* < –3) and leptokurtic (*kurtosis* >10) and were exponentially transformed (Fink, 2009) before they were analyzed. The transformed scores were within accepted limits (|*skew*|≤ 3 and |*kurtosis*|≤ 10; Kline, 2015).

### Hypothesis Testing: Adoption and Maintenance

H1-4 described predictors of adopting and maintaining the SET innovation: more positive attitudes about the innovation’s attributes, stronger descriptive norm of anticipated adoption, stronger tendency to adopt earlier, and sharing or hearing about the town hall meeting through WOM. To address nesting by village, we included a village code in the model (a code for treatment vs. control was not included because that information was not known at the time). To control for economic status, we also included economic resources as an independent variable. These hypotheses were tested simultaneously in separate ANCOVAs for adoption and maintenance (see Table 2). The two models were statistically significant: *F*(11, 317) = 8.34, *p* < .001, *R^2^* = .22 for intent to adopt; *F*(11, 317) = 7.43, *p* < .001, *R^2^* = .21 for intent to maintain.

**Table 2.** Estimates of predictors on intentions to adopt, maintain, and discuss the eave tubes

	Adoption			Maintenance			Talk		
	*β*	*b*	*SE*	*β*	*b*	*SE*	*β*	*b*	*SE*
Attribute attitudes	.13*	18.81	7.89	.15**	21.26	7.51	.62**	1.14	0.09
Descriptive norm	.23**	5.40	1.30	.18**	3.94	1.24	−.12*	−0.03	0.01
Earlier adopter	.07	1.83	1.68	−.05	−1.23	1.60	.12*	0.04	0.02
Town hall shared	−.05	−2.23	2.60	.06	2.84	2.47	.12*	0.07	0.03
Town hall WOM	−.20**	−9.27	2.44	−.02	−0.86	2.32	.05	0.03	0.03
Economic resources	.13*	4.05	1.62	.10	2.90	1.54	.03	0.01	0.02
*R*^2^	.22*			.21*			.41*		

*Note*. Parameters produced from ANCOVAs in which the village code and the predictors were included. All models were statistically significant: *F*(11, 317) = 8.34, *p* < .001, *R* ^2^ = .22 for intent to adopt; *F*(11, 317) = 7.43, *p* < .001, *R* ^2^ = .21 for intent to maintain; and *F*(11, 317) = 19.96, *p* < .001, *R* ^2^ = .41 for intent to talk.

* *p* < .05.

As predicted, property owners with more positive attitudes about the innovation’s attributes had stronger intentions to adopt and maintain the innovation. H1 was supported. Also as predicted, property owners with stronger descriptive norms had stronger intentions to adopt and to maintain the innovation; H2 was supported. Counter to prediction, the reported tendency to adopt innovation before others was unrelated to intentions to adopt or maintain the innovation; H3 was not supported. Counter to prediction, property owners who attended the town hall meeting and discussed it with others (versus those who did not) had similar intentions to adopt and maintain the innovation. Unexpectedly, property owners who heard about the town hall meeting from others (versus those who did not) had weaker intentions to adopt the SET innovation, but no difference in intentions to maintain them. H4 was not supported. Unexpectedly, it appears that hearing the WOM reports was detrimental.

### Adopting Earlier: Exploring Moderation

To explore whether early and late adopters differ in how much attitudes and norms predict their intention to adopt (RQ1), we recoded the continuous tendency of adopting earlier into two codes: early adopters (means greater than 4, *n* = 77) and later adopters (*n* = 252). In the separate samples, after controlling for village, attribute attitudes and norms were correlated with intention to adopt. The correlations were compared between samples using *z* tests. Counter to the prediction, the positive association between positive attribute attitudes and intention to adopt the innovation was consistent across groups: *r*(75) = .13 for early adopters, *r*(250) = .22, *z *= 0.69, *p* = .49. Counter to the prediction, the positive association between greater descriptive norms and greater intentions to adopt the innovation was stronger for early adopters, *r*(75) = .40, than later adopters, *r*(250) = .23; the difference was not statistically significant, *z* = 1.47, *p* = .14.

#### Town Hall Reports

To explore what was discussed in the WOM conversations about the town hall meeting (RQ3), we analyzed property owners’ reports of what they told people or what they heard about the town hall meeting. A codebook was developed based on theoretical constructs from the DOI (e.g., uncertainty, innovation attributes) and the IMB (e.g., attitude). In addition, a protocol category was defined for statements of fact about the trial, and a misinformation category was defined for incorrect statements about the trial. Last, two categories captured whether participants’ adoption was voluntary or compulsory (of note, trial participation was voluntary). The unit of analysis was a complete thought representing participants’ perception and understanding of the trial. For example, a response that “the eave tubes will prevent malaria, and make the house prettier” included two units. After training, the authors coded about 20% of the open-ended responses; the few disagreements were resolved through discussion. The two coders independently coded the rest of the data. Intercoder reliabilities ranged from .66 to 1. Disagreements about the final codes were resolved through discussion. The coding categories and examples appear in Table 3.

**Table 3.** Examples, frequency, and intercoder reliability of what was shared and heard about the town hall meeting through word of mouth

		Shared		Heard	
Coding category	Examples	*n*	*κ*	*n*	*κ*
Protocol	The Eave Tube project consists of installing eave tubes in the wall.	58	0.69	37	0.90
Misinformation	There is a new disease caused by mosquitos.	0	n/a	1	1.00
Novelty	The Eave Tube is a new method against malaria.	10	0.94	12	1.00
Uncertainty	What is the Eave Tube project?	0	n/a	2	1.00
Positive Attitude	Eaves Tube will be very good.	5	1.00	19	0.92
Relative Advantage	Eave Tube will better prevent malaria.	1	1.00	1	1.00
Compatibility	Our houses will look nice.	1	1.00	0	n/a
Complexity	Eave Tube is easy to use.	0	n/a	1	1.00
Compulsory	We have to adopt it.	5	0.88	10	0.87
Voluntary	We can accept the Eave Tube or not. It is not compulsory.	1	1.00	1	0.66

*Notes*. Three additional coding categories were used, including negative attitude, trialibility, and observability. These coding categories were not observed in the reports of what they said or heard, and were not included in the table.

The most common open-ended responses about the town hall meeting concerned the field trial’s protocol, such as bringing bed nets, drilling holes in the walls, putting up screening, and periodic visits from researchers and trial staff. The next most popular topic reported by those *sharing* information about the town hall meeting related to novelty, followed by positive attitudes about the innovation and a feeling that people needed to adopt it. For those reporting on what they *heard*, the next most popular topic after the trial’s protocol was positive attitudes about the innovation, its novelty, and the need to adopt it. Very few answers fit the explicit categories of innovation attributes, such as its relative advantage. It is also important to note what was not discussed: negative attitudes and the voluntary nature of participating in the trial. Misinformation or uncertainty was reported only by those people hearing the WOM, and these reports were rare.

### Hypothesis Testing: Social Diffusion

H5 predicted that more positive attribute attitudes, weaker descriptive norms, greater tendencies to be earlier adopters, and involvement in WOM conversations about the town hall meeting would predict greater intentions to talk about the SET innovation with friends and family. The same ANCOVA model used for predicting adoption and maintenance was used for talk, because the variables were the same for all of the models. The ANCOVA was statistically significant: *F*(11, 317) = 19.96, *p* < .001, *R^2^* = .41.

As predicted, more positive attribute attitudes predicted intentions to talk. Also as predicted, weaker descriptive norms predicted greater intention to talk. In addition, as predicted, sharing about the town hall meeting through WOM predicted greater intentions to talk, but, counter to prediction, hearing about it did not.

## Discussion

We integrated the DOI and the IMB to predict intentions to adopt, maintain, and talk about the SET innovation in a large field trial with potential end-users. The findings showed that positive attitudes about the innovation’s attributes were a consistent positive predictor of diffusion intentions: adopting, maintaining, and talking with others about it. As expected by the DOI and the IMB, the social pressure created by descriptive norms (i.e., anticipating that more people in the community, including leaders, would adopt the innovation) positively predicted intentions to adopt and to maintain the SET innovation. Drawing upon social-transmission research (Berger, 2014; Cappella et al., 2015), we argued that the descriptive norm may dampen future talk about it, because it may no longer be seen as a novel, useful topic to discuss. As predicted, the results showed that as the descriptive norm increased, the intention to talk about the innovation decreased. These results provide broad support for integrating the DOI and the IMB in efforts to predict diffusion and highlight the need to draw on other research to understand motivations for social diffusion.

We also explored the personal tendency to adopt innovations before others. Earlier adopter tendencies did not have a direct effect on intentions to adopt or maintain the SET innovation. The moderation tests showed a surprising trend countering the prediction that earlier adopters are motivated by attitudes and later adopters by social pressures. The relationship between descriptive norms and adoption was almost twice as strong for earlier adopters than for later adopters. This finding aligned Rogers’s (2003) argument that opinion leaders tend to adopt innovations before others but are not the most innovative. Opinion leaders maintain the respect and credibility of their followers by carefully judging the innovation’s risk and the community’s norms. If the public adopts a low-risk innovation, an opinion leader can lose credibility by not adopting; conversely, if a leader who adopts a risky innovation can lose credibility.

The biggest surprise was that participants who heard about the town hall meeting from others through WOM had weaker intentions to adopt the innovation. Participants’ open-ended responses about what they heard or shared with others about the town hall meeting revealed a few possible reasons. First, although the most common topic was the field trial’s protocol (e.g., drilling holes in the walls), the WOM content left out important features and functions of the technology, such as why and how it works to prevent exposure to infectious mosquitos. Although information loss due to memory limits is normal during information sharing (Bartlett, 1932), the absence of understanding how and why the innovation works may lead to misunderstandings about the innovation, concerns about whether it is working, and whether it is safe. The novelty of this innovation must be justified to participants, or it may come across as shocking, which may increase uncertainty. For those reporting on what they heard through WOM, the most popular topic after the trial’s protocol was positive attitudes about the innovation, its novelty, and the need to adopt it. Reports of hearing the need to adopt may reveal another reason: if the listener perceived this as an attempt to influence them to adopt, they may have felt less freedom to choose. People can react to freedom-threatening messages by not engaging in the recommended act in order to restore freedom (Brehm, 1966).

### Limitations

The sample was limited to potential adopters: property owners of suitable homes. It is unclear to what extent those who could not adopt may influence community support for an innovation. Other studies find that those who are not eligible to adopt still intend to persuade others who can to act (Smith & Carpenter, 2017). Also, although self-efficacy was less relevant to the field-testing phase, long-term success would require maintenance; the effects of maintenance self-efficacy and potential costs should be considered in future research.

### Implications for Practice

Our results showed that stronger social pressures with regards to descriptive norms were positively linked to people’s intention to adopt and to maintain the eave tubes, but were negatively associated with intention to talk about them. Campaigns that use descriptive norms as a means to promote diffusion face a dilemma: over-promoting community acceptance before it occurs may dampen conversations about it, thus ultimately dampening diffusion. This finding highlights the importance of considering the social context where a campaign is implemented. It also emphasizes the need to track the content of conversations. This call is not new (Southwell & Yzer, 2009). Southwell (2017) noted that many studies on diffusion and campaign evaluation treated conversation as “a monolithic entity that can be counted” (p. 229). From the current data, it is clear that conversational content is diverse and complex: it may contain laudatory as well as disparaging information, and it may contain truthful and inaccurate information.
